# Mediterranean diet and risk of rheumatoid arthritis: a population-based case-control study

**DOI:** 10.1186/s13075-018-1680-2

**Published:** 2018-08-09

**Authors:** Kari Johansson, Johan Askling, Lars Alfredsson, Daniela Di Giuseppe, on behalf of the EIRA study group

**Affiliations:** 1 Department of Medicine, Solna, Clinical Epidemiology Division, Karolinska Institutet, SE-171 76 Stockholm, Sweden; 2 Department of Medicine, Solna, Rheumatology Division, Karolinska Institutet, SE-171 76 Stockholm, Sweden; 3 Institute of Environmental Medicine, Karolinska Institutet, Stockholm, Sweden; 4 Centre for Occupational and Environmental Health, Stockholm County Council, Stockholm, Sweden

**Keywords:** Mediterranean diet, Rheumatoid arthritis, Rheumatoid factor, ACPA, Smoking

## Abstract

**Background:**

The Mediterranean diet has been associated with lower mortality and lower risk of cardiovascular diseases and cancer. Although its components have been analysed in several studies, only one study has specifically investigated the association between Mediterranean diet and risk of rheumatoid arthritis (RA), and reported no association.

**Methods:**

Data on 1721 patients with incident RA (cases) and 3667 controls, matched on age, gender and residential area, from the Swedish epidemiological investigation of RA (EIRA), a population-based case-control study, were analysed using conditional logistic regression. The Mediterranean diet score, ranging from 0 to 9, was calculated from a 124-item food frequency questionnaire.

**Results:**

In the EIRA study (median age of participants 53 years), 24.1% of the patients and 28.2% of the controls had high adherence to the Mediterranean diet (a score between 6 and 9). After adjustments for body mass index, educational level, physical activity, use of dietary supplements, energy intake, and smoking, high adherence reduced the odds of developing RA by 21% (OR 0.79; 95% CI 0.65–0.96) as compared to low adherence (a score between 0 and 2). The OR was even lower among men (OR 0.49; 95% CI 0.33–0.73), but no significant association was found among women (OR 0.94; 95% CI 0.74–1.18). An association between high diet score and low risk of RA was observed in rheumatoid factor (RF)-positive (OR 0.69; 95% CI 0.54–0.88), but not RF-negative RA (OR 0.96; 95% CI 0.68–1.34), and in RA characterised by presence of antibodies to citrullinated peptides (ACPA), but not in ACPA-negative RA.

**Conclusions:**

In this large population-based case-control study, the Mediterranean diet score was inversely associated with risk of RA. However, an association was only found among men and only in seropositive RA.

**Electronic supplementary material:**

The online version of this article (10.1186/s13075-018-1680-2) contains supplementary material, which is available to authorized users.

## Background

Rheumatoid arthritis (RA) is a chronic inflammatory autoimmune disease. The aetiology includes complex genetics and interplay with environmental factors. In contrast to the growing number of genetic loci associated with RA risk, the number of identified modifiable risk factors is still limited, which in turn limits the potential for primary prevention. Smoking has been shown to increase the risk of RA [1], while a moderate alcohol intake has been associated with decreased risk of RA [2, 3]. Consumption of fish (especially fish rich in long-chain n-3 polyunsaturated fatty acids) [4, 5] and physical activity have both been linked to potentially decreased risks of RA [6, 7].

The Mediterranean diet is foremost a plant-based diet including high consumption of fruit, vegetables, whole grains and legumes, moderate consumption of fish, white meat and alcohol and low consumption of red meat and sugar. The diet is characterised by a high ratio of monounsaturated to saturated fats with total fat accounting for 30–40% of daily energy consumption [8]. The Mediterranean diet has been associated with lower risk of overall mortality, cardiovascular disease and cancer [9–11]. Due to its presumed anti-inflammatory properties [12] the Mediterranean diet might reduce the risk of inflammatory conditions, such as RA. So far, however, only some of its components have been studied, while little is known about the potential link between the Mediterranean dietary pattern and risk of RA. Only one previous study, a cohort study of women only from the Nurses’ health study (NHS) in the USA [13], have specifically investigated the association between the Mediterranean diet and risk of RA. Another study, a nested case-control study from Sweden [14], has also analysed the effect the Mediterranean diet, but not as the main outcome. Neither study found any association between the Mediterranean diet and risk of RA. However, the NHS only included women, and the Swedish study did not investigate the effect in men and women separately.

The aim of this study was therefore to investigate the association between the Mediterranean diet and risk of RA, taking RA subtype, gender and other RA risk factors into account.

## Methods

### Study population

This study included subjects from the Swedish epidemiological investigation of RA (EIRA), a population-based case-control study, from defined geographical areas of central Sweden. The EIRA study was initiated in 1996 and is still ongoing. The general study design has been described in detail elsewhere [15]. Briefly, the study includes patients with incident RA (cases) (mean time between symptom onset and diagnosis 9.7 months) diagnosed by a rheumatologist according to the either the American College of Rheumatology (ACR) 1987 criteria or the 2010 ACR/European League Against Rheumatism (EULAR) classification criteria for RA. To each patient two controls were randomly selected from the register of the general population, in connection to inclusion of the patient, matched on age, sex and residential area.

A baseline questionnaire was distributed to patients and controls with comprehensive questions on lifestyle factors, educational level and comorbidities. From November 2005 the EIRA questionnaire also included a 124-item food frequency questionnaire (FFQ). The current study therefore included participants from November 2005 to September 2014.Participants with incomplete an FFQ where excluded (*n* = 21).

Participants provided written informed consent, and ethical approval was obtained from the Regional Ethical Review Board at Karolinska Institutet, Stockholm, Sweden.

### Exposure definition

The self-reported FFQ was used to evaluate the participants’ food intake during the last year before inclusion. In the FFQ participants were asked to indicate how often on average they had consumed various foods by using eight predefined frequency categories ranging from “never or less than once per month” to “three or more times per day”. Fat and energy intake was calculated by multiplying the average frequency of consumption of each food by the fat and energy content, respectively, of age-specific portion sizes, according to the Swedish National Food Administration Database [16].

The Mediterranean diet was defined according to the Mediterranean diet score based on the work of Trichopoulou et al. [17]. The score range between 0 and 9 and each person was assigned 1 point if she/he consumed more than the sex-specific median of the population of the beneficial components, such as vegetables, legumes, fruits and nuts, cereal and fish, or if she/he consumed less than the median of red meat and dairy products, which were considered detrimental components. Moreover, 1 point was assigned to men who consumed between 10 g and 50 g of alcohol per day and to women who consumed between 5 g and 25 g per day, and 1 point if the ratio of monounsaturated lipids to saturated lipids was above the median. Hence, a higher score corresponded to higher adherence to the Mediterranean diet. We did not include butter among the dairy products since it was reported in the FFQ only as “ever consumption”. In sensitivity analysis, we considered ever consumption of butter as consumption once per day and added it to the dairy products group.

Modifications to the original Mediterranean score have been proposed throughout the years [18, 19]. In sensitivity analyses, we defined the Mediterranean diet according to the alternate Mediterranean diet score (aMed), which not include dairy products in the score [13]. Moreover, we used a lower upper limit for alcohol intake among men (30 g instead of 50 g per day), according to the modified Mediterranean diet (mMED) score [19].

### Covariate definition

All covariate data were collected at baseline. Smoking was categorised as never, current, former or irregular smoker. Body mass index (BMI) was categorised as underweight (< 18.5 kg/m^2^), normal weight (18.5–24.9 kg/m^2^), overweight (25–29.9 kg/m^2^) or obese (≥ 30 kg/m^2^). Education was categorised as <10 years, 10–12 years, and > 12 years of education. Physical activity was reported as sedentary, moderate, moderate/regular, exercise/regular. Nutritional supplements were defined as intake in the last 5 years of at least one of the following supplements: multivitamins (with or without minerals), vitamin C, vitamin E, vitamin B, vitamin A, vitamin D, calcium, zinc, iron, magnesium, folic acid, β-caroten or omega 3. Additional covariates were considered when analysing women only: parity (quartiles), age at menarche (quartiles), use of oral contraceptive (yes/no) and use of hormone replacement therapy (yes/no). Genotyping of the *HLA-DRB1* gene was conducted as previously described [20, 21]. Among *HLA-DRB1* genes, DRB1*01, DRB1*04 and DRB1*10 genes were defined as SE alleles. Any genotype containing one or two of these genes was considered as having “any SE allele” versus those not having any of the genes (“no SE alleles”).

### Statistical analyses

The Mediterranean diet score was analysed both as a continuous and as a categorical variable, the latter according to the quartiles of the distribution. Odds ratios (ORs) and their 95% confidence intervals (CIs) were calculated using conditional logistic regression, and multivariable models were adjusted for smoking, BMI, educational level, physical activity, use of dietary supplements, and energy intake. Analyses were stratified by gender, rheumatoid factor (RF) and anti-citrullinated protein antibody (ACPA) status. We additionally stratified by smoking status and presence of at least one copy of the HLA-DRB1 shared epitope allele. In sensitivity analyses, we excluded persons with extreme energy intake (i.e. 3 standard errors from the mean value on the log-transformed scale, *n* = 49). Moreover, to evaluate the influence of each single item, we analysed data excluding one component of the score at a time.

We estimated the dose–response trend association between the Mediterranean diet score and risk of RA using restricted cubic splines with knots at 10, 50 and 90 centiles of the Mediterranean score (e.g. at 2, 4, and 7) [22].

To analyse the association in a group of patients presumed to have more homogeneous disease aetiology, we restricted the analyses to patients and controls with at least one copy of the HLA-DRB1 shared epitope allele (SE) and who were also current smokers. For this analysis, since patients and controls were no longer matched, we performed logistic regression adjusted for age, gender and residential area, and then additionally adjusted for the confounding variables listed above. Statistical analyses were implemented using SAS (V.9.4) and Stata (V.13.1), and *p* values ≤0.05 were considered significant.

### Patient involvement

There was no patient involvement in the current study.

## Results

### Study characteristics

This study included 1721 patients with RA (cases) and 3667 controls from the EIRA study. Table 1 presents the baseline characteristics of patients with RA and controls overall and by categories of the Mediterranean diet, with high adherence to the Mediterranean diet score ranging between 6 and 9, and a low score between 0 and 2. Patients with RA were more often smokers, more sedentary and consumed more nutritional supplements, as compared with the controls. Total energy intake was, however, similar between the groups, and when assessed separately by food item category, the servings per day were also similar between patients and controls, except for alcohol consumption, which was slightly lower among patients (7.1 g per day) versus controls (8.5 g per day). There were no differences between women and men or by RA subtype in terms of intake of individual nutrient components, except for alcohol intake (Additional file 1: Table S1–S4).

**Table 1:** Baseline characteristics of patients with RA (cases, *n* = 1721) and controls (*n* = 3667) in the EIRA study, overall and by categories of the Mediterranean diet score as defined according to Trichopoulou et al. [17]

Med score	RA cases					Controls				
	Overall	0–2	3	4–5	6–9	Overall	0–2	3	4–5	6–9
Number	1721	351	327	629	414	3667	574	636	1424	1033
Female, *n* (%)	1251 (72.7)	228 (65.0)	242 (74.0)	458 (72.8)	323 (78.0)	2647 (72.2)	411 (71.6)	441 (69.3)	1026 (72.1)	769 (74.4)
Age, mean (std)	53.4 (13.5)	53.1 (14.5)	52.2 (14.3)	53.1 (13.6)	55.2 (11.4)	53.3 (13.3)	51.1 (14.8)	53.2 (13.6)	53.5 (13.0)	54.5 (12.6)
Current smoking, *n* (%)	409 (23.8)	125 (35.6)	92 (28.1)	132 (20.0)	60 (14.5)	543 (14.8)	122 (21.3)	116 (18.2)	210 (14.8)	95 (9.2)
Sedentary physical activity, *n* (%)	317 (18.4)	78 (22.2)	79 (24.2)	109 (17.3)	51 (12.3)	458 (12.5)	110 (19.2)	94 (14.8)	161 (11.3)	93 (9.0)
Education > 12 years, *n* (%)	955 (55.5)	150 (42.7)	176 (53.8)	345 (54.9)	284 (68.6)	2161 (58.9)	279 (48.6)	336 (52.8)	859 (60.3)	687 (66.5)
BMI, mean (std)	25.7 (4.6)	25.5 (4.9)	25.6 (4.3)	26.1 (4.7)	25.3 (4.5)	25.4 (5.0)	25.6 (4.8)	25.6 (4.2)	25.5 (4.2)	25.1 (6.4)
Use of supplements, *n* (%)	858 (50.1)	153 (44.1)	146 (44.8)	316 (50.3)	243 (58.8)	1616 (44.4)	214 (37.7)	260 (41.1)	624 (44.1)	518 (50.5)
Total energy intake, mean kcal/day (std)	1924 (735)	1848 (721)	1829 (730)	1949 (824)	2025 (574)	1897 (719)	1770 (763)	1776 (684)	1892 (743)	2048 (652)
Vegetables, servings/day (std)	3.07 (2.00)	1.60 (0.99)	2.41 (1.57)	3.26 (1.80)	4.53 (2.12)	3.22 (2.24)	1.78 (1.17)	2.25 (1.59)	3.22 (2.16)	4.61 (2.31)
Legumes, servings/day (std)	0.22 (0.26)	0.10 (0.14)	0.14 (0.15)	0.23 (0.24)	0.38 (0.33)	0.24 (0.31)	0.09 (0.09)	0.13 (0.16)	0.23 (0.27)	0.41 (0.41)
Fruit and nuts, servings/day (std)	1.57 (1.39)	0.76 (0.70)	1.11 (1.16)	1.61 (1.23)	2.53 (1.62)	1.67 (1.42)	0.82 (0.77)	1.15 (1.06)	1.69 (1.43)	2.42 (1.49)
Cereals, servings/day (std)	2.78 (1.90)	2.04 (1.76)	2.63 (1.88)	2.90 (1.91)	3.36 (1.79)	2.72 (1.83)	2.01 (1.71)	2.48 (1.86)	2.73 (1.82)	3.23 (1.73)
Fish, servings/day (std)	0.48 (0.44)	0.28 (0.16)	0.36 (0.23)	0.51 (0.61)	0.68 (0.33)	0.49 (0.38)	0.28 (0.20)	0.36 (0.23)	0.47 (0.39)	0.69 (0.40)
Meat, servings/day (std)	1.28 (0.73)	1.24 (0.72)	1.28 (0.66)	1.31 (0.75)	1.25 (0.75)	1.28 (0.83)	1.28 (0.72)	1.25 (0.74)	1.33 (0.96)	1.25 (0.74)
Dairy products, servings/day (std)	3.30 (2.49)	4.63 (2.35)	3.57 (2.48)	3.15 (2.48)	2.20 (2.03)	3.03 (2.41)	4.32 (2.39)	3.58 (2.62)	2.90 (2.31)	2.16 (1.98)
Alcohol, g/day (std)	7.06 (7.95)	5.15 (7.94)	6.98 (8.28)	7.28 (7.97)	8.38 (7.37)	8.48 (8.70)	6.20 (9.28)	7.95 (9.27)	8.54 (8.60)	9.94 (7.85)
Monoansaturated to saturated fat ratio (std)	0.78 (0.14)	0.69 (0.09)	0.74 (0.12)	0.80 (0.14)	0.86 (0.15)	0.79 (0.15)	0.70 (0.10)	0.74 (0.13)	0.80 (0.14)	0.87 (0.15)

*RA* rheumatoid arthritis, *Med score* Mediterranean diet score, *std* standard deviation, *BMI* body mass index

### Mediterranean diet and risk of RA

Fewer patients with RA than controls had high adherence (score 6–9) to the Mediterranean diet (patients 24.1%, controls 28.2%). This pattern was similar among men and women, but women were more often in the highest category of the score (among patients, women 25.8%, and men 19.4%; among controls, women 29.1% and men 25.9%).

**Fig. 1:**
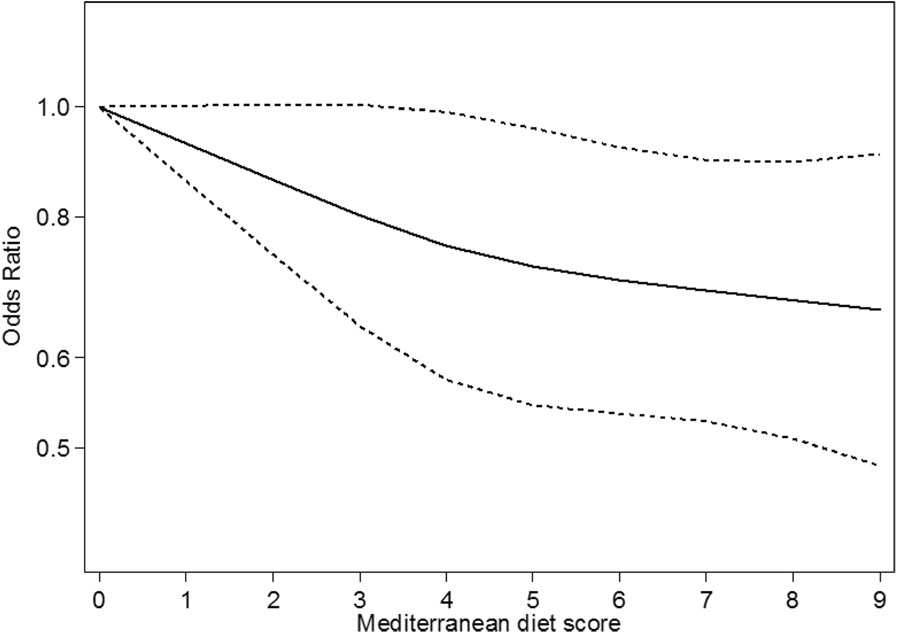
Dose-response odds ratio for risk of rheumatoid arthritis (RA) by the Mediterranean diet score as defined according to Trichopoulou et al. [17] among 1721 patients with RA and 3667 controls. Adjusted for smoking, physical activity, education level, body mass index (BMI), energy intake and use of supplements. Dotted lines correspond to 95% confidence intervals

The Mediterranean diet was associated with a decrease in the odds of developing RA (Fig. 1). One unit increase in the score corresponded to an 8% decrease in the odds of having RA (crude OR conditioned on matching factors = 0.92, 95% CI 0.89–0.95). After additional adjustment for smoking, physical activity, education level, BMI, energy intake and use of supplements, the OR was 0.95 (95% CI 0.92–0.99). The restricted cubic splines model showed a dose–response trend with no departure from linearity (i.e. the second coefficient of the spline was not statistically significant).

High adherence to the Mediterranean diet (score 6–9) reduced the odds of developing RA by 34% (OR = 0.66, 95% CI 0.55–0.79) as compared to low adherence (score 0–2), based on the crude model conditioned on matching factors. The association was somewhat attenuated after adjustment for covariates, yet remained statistically significant, OR = 0.79 (95% CI 0.65–0.96) (Table 2).

**Table 2:** Odds ratios of rheumatoid arthritis by categories of the Mediterranean diet score among participants of the EIRA study (*n* = 1721 patients with RA (cases) and *n* = 3667 controls)

	Mediterranean-diet score			
	0–2	3	4–5	6–9
Overall				
Number	351/574	327/636	629/1424	414/1033
OR crude	Ref	0.87 (0.71–1.05)	0.74 (0.62–0.87)	0.66 (0.55–0.79)
OR adjusted	Ref	0.93 (0.76–1.14)	0.83 (0.69–0.99)	0.79 (0.65–0.96)
Female				
Number	228/411	242/441	458/1026	323/769
OR crude	Ref	1.01 (0.80–1.28)	0.81 (0.66–0.99)	0.77 (0.62–0.95)
OR adjusted	Ref	1.09 (0.85–1.39)	0.92 (0.75–1.14)	0.94 (0.74–1.18)
OR adjusted 2	Ref	1.07 (0.84–1.37)	0.91 (0.73–1.12)	0.94 (0.74–1.18)
Male				
Number	123/167	84/195	171/398	91/264
OR crude	Ref	0.61 (0.43–0.87)	0.59 (0.43–0.80)	0.45 (0.32–0.64)
OR adjusted	Ref	0.65 (0.45–0.95)	0.62 (0.45–0.87)	0.49 (0.33–0.73)
RF positive				
Numbe	238/327	232/356	401/841	248/627
OR crude	Ref	0.90 (0.70–1.14)	0.66 (0.54–0.81)	0.55 (0.44–0.69)
OR adjusted	Ref	0.97 (0.75–1.25)	0.76 (0.61–0.95)	0.69 (0.54–0.88)
RF negative				
Number	113/184	90/205	226/422	163/307
OR crude	Ref	0.77 (0.55–1.08)	0.91 (0.68–1.21)	0.89 (0.66–1.21)
OR adjusted	Ref	0.80 (0.56–1.14)	0.93 (0.68–1.27)	0.96 (0.68–1.34)
ACPA positive				
Number	247/343	240/387	424/863	264/646
OR crude	Ref	0.87 (0.69–1.10)	0.68 (0.56–0.83)	0.58 (0.47–0.72)
OR adjusted	Ref	0.95 (0.74–1.21)	0.78 (0.63–0.97)	0.72 (0.57–0.92)
ACPA negative				
Number	103/170	84/176	203/403	149/279
OR crude	Ref	0.84 (0.58–1.20)	0.88 (0.65–1.19)	0.91 (0.66–1.26)
OR adjusted	Ref	0.89 (0.61–1.30)	0.93 (0.67–1.29)	1.00 (0.70–1.42)

Odds ratio (OR) crude was conditioned on age, gender and residential area. OR adjusted was adjusted for smoking, physical activity, education level, body mass index, energy intake and supplements use. OR adjusted 2 was additionally adjusted for parity, use of oral contraceptive, use of hormone replacement therapy and age at menarche

*RA* rheumatoid arthritis, *ACPA* antibodies to citrullinated peptides, *Ref* reference

When stratified by gender, the inverse association between high adherence to the Mediterranean diet and RA was statistically significant among men (OR = 0.49, 95% CI 0.33–0.73), but not among women (OR = 0.94, 95% CI 0.74–1.18). Adding further covariates for women (parity, use of oral contraceptive, use of hormone replacement therapy, age at menarche), did not change the results. A multiplicative interaction between high adherence to the Mediterranean diet and gender was found (*p* < 0.01).

When stratified by RA subtype, the inverse association between Mediterranean diet score and RA was observed only among seropositive patients. A high score corresponded to lower odds in RF-positive but not RF-negative patients and in ACPA-positive but not ACPA-negative patients. When stratifying by RA subtype and gender, we found that a high Mediterranean diet score was associated with a non-significantly reduced risk of RF-positive RA among women (adjusted OR (ORadj) = 0.81, 95% CI 0.61–1.07), and was similar in ACPA-positive RA (ORadj = 0.85, 95% CI 0.64–1.13), while among men there was a reduced risk in both RF-positive (ORadj = 0.44, 95% CI 0.26–0.74) and ACPA-positive RA (ORadj = 0.46, 95% CI 0.28–0.76).

In the subset-analyses of patients with RA who were smokers and who carried the HLA-DRB1 SE allele (*n* = 266), compared to controls who were also smokers and carried the HLA-DRB1 SE allele (*n* = 121) (Table 3), a high score was associated with a 37% lower odds of developing RA, although this was not statistically significant (adjOR = 0.63, 95% CI 0.28–1.37).

**Table 3:** Odds ratios for rheumatoid arthritis (RA) by categories of the Mediterranean diet score among participant in the EIRA study who were current smokers and carrying at least one HLA-DRB1 shared epitope allele (*n* = 266 patients with RA (cases) and *n* = 121 controls)

	Mediterranean diet score			
	0–2	3	4–5	6–9
Overall				
Number	83/23	61/29	79/48	43/21
OR crude	Ref	0.61 (0.31–1.18)	0.45 (0.25–0.83)	0.57 (0.27–1.18)
OR adjusted	Ref	0.61 (0.31–1.22)	0.53 (0.28–1.02)	0.63 (0.28–1.37)

Odds ratio (OR) crude was conditioned on age, gender and residential area. OR adjusted was adjusted for smoking, physical activity, education level, body mass index, energy intake and use of supplements

*Ref* reference

Similar results, although statistically significant, were found when analysing smokers separately irrespectively of HLA-DRB1 SE allele status (adjOR = 0.62, 95% CI 0.40–0.95, Additional file 1: Table S5). Also, analysing only those with the HLA-DRB1 SE allele similar results were retrieved (adjOR = 0.60, 95% CI 0.44–0.82; Additional file 1: Table S6).

### Sensitivity analysis

We excluded one component of the score at a time. The inverse association with RA observed for the highest category of the score ranged between 0.74 (95% CI 0.60–0.91) after exclusion of the fat-ratio group to 0.85 (95% CI 0.70–1.04) after exclusion of alcohol.

Alternative definitions of the diet score had little impact on the observed association. Inclusion of butter did not change the results (OR for a score of 6–9 vs. 0–2 = 0.79, 95% CI 0.64–0.96). When persons with extreme energy intake (i.e. 3 standard errors from the mean value on the log-transformed scale, *n* = 49) were excluded, the results did not change (OR for a score of 6–9 vs. 0–2 = 0.79, 95% CI 0.65–0.96). We considered a different definition of moderate alcohol consumption for men: we lowered the upper limit of 50 g per day to 30 g per day. The results did not change overall (OR = 0.80, 95% CI 0.66–0.97) nor for men specifically (OR = 0.51, 95% CI 0.35–0.75). Moreover, we applied the aMed score, resulting in an association between the Mediterranean diet and RA that was no longer statistically significant, either overall (OR = 0.83, 95% CI 0.63–1.09) or in men (OR = 0.78, 95% CI 0.43–1.41).

## Discussion

### Main findings

In this large population-based case-control study, we observed that the Mediterranean diet score was inversely associated with risk of RA. However, this association was observed only among men and among individuals with seropositive RA, and could not readily be explained by other risk factors. A tendency toward lower risk of RA was found among smokers who carried the HLA-DRB1 shared epitope allele and who had a high Mediterranean diet score.

### Previous research

Previous studies of diet and risk of RA have been focused foremost on individual components, and the main dietary compounds that have been linked to a potentially decreased risk of RA include moderate alcohol intake and [2, 3] and consumptions of fish [4, 5], both of which are components of the Mediterranean diet. Currently, only one prospective cohort study from the NHS (pooled data from NHS I and II) has specifically examined the association between the Mediterranean diet and risk of RA [13]. This study included 174,638 female nurses and identified 913 incident cases of RA over 20 years of follow up [13]. Similar to our result among women, that study did not find any overall association between the Mediterranean diet and risk of RA. A nested case-control study in the Swedish Västerbotten intervention programme (a prospective population-based study [14]) investigated the effect of diet, looking at food groups, macronutrients and dietary pattern, including the Mediterranean diet. This study comprised 386 incident RA cases and 1886 matched controls and did not find an association between the Mediterranean diet and RA. Similar to our current study, the majority of the participants were women (70%), but no subgroup analysis by sex was performed.

Sundström et al. [14] used the Mediterranean-diet score of Trichopoulou et al. [17] to define the Mediterranean diet, as we did in the current study, while Hu et al. [13] used a modified version of the score (aMed score) [18]. The main differences between the original Mediterranean diet score [17] and the modified version [18] are the exclusion of dairy products from the aMed score and the different definition of moderate alcohol intake. In the original score moderate alcohol consumption was defined as 10–50 g per day for men and 5–25 g per day for women, whereas in the aMed it is defined as 10–15 g per day for men and 5–15 g per day for women. Indeed, when we applied the aMed score in our study, the association was no longer significant. Similarly, when we excluded one item of the score at a time, the association was no longer statistically significant when alcohol was excluded from the analyses, thus implying an influence of alcohol consumption in the prevention of RA, as shown elsewhere [3, 23].

### Strengths and limitations

Although the EIRA study is one of the largest population-based case-control studies including incident cases of RA, with extensive information, making it possible to account for many potential confounders, there are limitations of this study. First, the exposure was self-reported via a single-time-administered FFQ assessing dietary intake 1 year prior to RA diagnosis (at inclusion for controls). Although the FFQ used in this and other studies has proven to have high validity [24–26], any case-control study based on self-report may be prone to recall bias. Reverse causation may also be present as patients with early symptoms of RA might already have changed their diet during the year before diagnosis. However, the FFQ was administered close to onset of the first symptom of RA (mean duration 9.7 months). Moreover, a recent Swedish study suggested that that patients newly diagnosed with RA did not make any major dietary modifications after their diagnosis [27]. Another limitation is that the Mediterranean diet score (as well as other ranking scores) does not reflect the absolute Mediterranean diet, but the distribution of the score 0–9 in the studied population. This means that two persons with exactly the same nutritional intake but from two different study populations might end up with different scores. Moreover, even within the same study populations, the scores for women and men are calculated differently. Despite the extensive details of variables and potential confounders included in the EIRA study, there still might be residual confounding.

### Mechanisms

Although the Mediterranean diet has been shown to be effective in reducing overall mortality, cardiovascular disease and cancer [9–11], the biological mechanism is not fully understood. Potential mechanisms include lowering of blood pressure, lipids and inflammatory markers, via bioactive compounds that are rich in the Mediterranean diet, including antioxidants, monounsaturated and omega-3 fatty acids, phytosterols and fibre [28, 29].

In the current study, we found an inverse association between the Mediterranean diet and risk of RA but only among men, and only in seropositive RA. A difference in the effect of Mediterranean diet by gender has been shown before for cardiometabolic variables [30, 31], suggesting that indeed men could be more likely to be beneficially influenced by the Mediterranean diet. Moreover, differences in the aetiology of seropositive and seronegative RA have been shown before, in association with both genetic [32] and environmental risk factors [23, 33].

## Conclusion

We found that the Mediterranean diet score reduced the risk of RA; however, this was only among men and in seropositive RA. These results add to the accumulating evidence showing the importance of diet in the primary prevention of RA. However, we need to acknowledge that the mechanisms and impact of potential dietary guidelines might have to differ between RA sub-groups.

## Additional file

Additional file 1: Sensitivity analyses's tables. (DOCX 32 kb)

## Abbreviations

ACPA: Antibodies to citrullinated peptides
ACR: American College of Rheumatology
aMED: Alternate Mediterranean diet
BMI: Body mass index
CI: Confidence intervals
EIRA: Epidemiological investigation of rheumatoid arthritis
FFQ: Food Frequency Questionnaire
mMED: Modified Mediterranean diet
RA: Rheumatoid arthritis
RF: Rheumatoid factor
SE: Shared epitope
