# Supplementary material for: Mediterranean diet and risk of rheumatoid arthritis: a population-based case-control study
Source: Arthritis Res Ther. 2018 Aug 9;20:175. doi: 10.1186/s13075-018-1680-2 (PMC6085628; doi:10.1186/s13075-018-1680-2)
Supplement: Supplementary file 1 — Sensitivity analyses's tables. (DOCX 32 kb) [file 13075_2018_1680_MOESM1_ESM.docx]

**Supplemental Appendix to “Mediterranean diet and risk of rheumatoid arthritis: a population-based case-control study”**

**Table S1. Baseline characteristics of female cases (n=1251) and controls (n=2647) participating to the EIRA study, by categories of the Mediterranean-diet score.**

|  | **RA cases** | | | | | | | **Controls** | | | |  |
| --- | --- | --- | --- | --- | --- | --- | --- | --- | --- | --- | --- | --- |
| **Med Score** | **0-2** | | **3** | | **4-5** | | **6-9** | **0-2** | **3** | **4-5** | **6-9** | |
| **N** | 228 | 242 | | 458 | | 323 | | 411 | 441 | 1026 | 769 | |
| **Vegetables,servings/day** | 1.75 (1.02) | 2.60 (1.59) | | 3.44 (1.75) | | 4.80 (2.17) | | 1.92 (1.13) | 2.45 (1.71) | 3.40 (2.18) | 4.96 (2.43) | |
| **Legumes, servings/day** | 0.09 (0.14) | 0.13 (0.13) | | 0.24 (0.25) | | 0.40 (0.35) | | 0.09 (0.09) | 0.13 (0.16) | 0.23 (0.28) | 0.43 (0.44) | |
| **Fruit and nuts, servings/day** | 0.85 (0.72) | 1.18 (1.02) | | 1.73 (1.29) | | 2.72 (1.58) | | 0.93 (0.82) | 1.30 (1.13) | 1.82 (1.43) | 2.63 (1.51) | |
| **Cereals, servings/day** | 2.04 (1.63) | 2.47 (1.65) | | 2.75 (1.72) | | 3.12 (1.58) | | 2.02 (1.62) | 2.40 (1.73) | 2.61 (1.67) | 3.09 (1.60) | |
| **Fish, servings/day** | 0.29 (0.17) | 0.39 (0.23) | | 0.50 (0.31) | | 0.68 (0.31) | | 0.29 (0.21) | 0.39 (0.24) | 0.48 (0.32) | 0.70 (0.41) | |
| **Meat, servings/day** | 1.20 (0.71) | 1.24 (0.65) | | 1.25 (0.73) | | 1.23 (0.76) | | 1.24 (0.66) | 1.21 (0.72) | 1.26 (0.71) | 1.19 (0.72) | |
| **Dairy products, servings/day** | 4.44 (2.19) | 3.36 (2.39) | | 3.05 (2.40) | | 2.25 (2.10) | | 4.33 (2.35) | 3.57 (2.56) | 2.85 (2.29) | 2.15 (1.96) | |
| **Alcohol, g/day** | 4.35 (7.08) | 6.52 (8.26) | | 6.43 (7.32) | | 7.45 (6.53) | | 5.70 (9.37) | 7.19 (8.92) | 7.62 (7.86) | 9.00 (7.02) | |
| **Monoansaturated to saturated fat ratio** | 0.69 (0.09) | 0.74 (0.12) | | 0.79 (0.14) | | 0.86 (0.15) | | 0.68 (0.09) | 0.74 (0.13) | 0.79 (0.14) | 0.87 (0.16) | |

**Table S2. Baseline characteristics of male cases (n=470) and controls (n=1020) participating to the EIRA study, by categories of the Mediterranean-diet score.**

|  | **RA cases** | | | | **Controls** | | | |
| --- | --- | --- | --- | --- | --- | --- | --- | --- |
| **Med Score** | 0-2 | 3 | 4-5 | 6-9 | 0-2 | 3 | 4-5 | 6-9 |
| **N** | 123 | 85 | 171 | 91 | 163 | 195 | 398 | 264 |
| **Vegetables, servings/day** | 1.32 (0.87) | 1.85 (1.36) | 2.77 (1.86) | 3.58 (1.63) | 1.43 (1.19) | 1.78 (1.14) | 2.75 (2.03) | 3.61 (1.53) |
| **Legumes, servings/day** | 0.10 (0.13) | 0.15 (0.20) | 0.19 (0.22) | 0.32 (0.25) | 0.08 (0.08) | 0.12 (0.16) | 0.20 (0.25) | 0.34 (0.30) |
| **Fruit and nuts, servings/day** | 0.59 (0.63) | 0.93 (1.49) | 1.29 (0.96) | 1.89 (1.61) | 0.56 (0.54) | 0.81 (0.80) | 1.35 (1.36) | 1.83 (1.23) |
| **Cereals, servings/day** | 2.03 (1.99) | 3.07 (2.37) | 3.32 (2.30) | 4.18 (2.20) | 2.00 (1.92) | 2.67 (2.13) | 3.05 (2.13) | 3.67 (2.00) |
| **Fish, servings/day** | 0.25 (0.15) | 0.29 (0.18) | 0.53 (1.04) | 0.69 (0.40) | 0.23 (0.15) | 0.31 (0.18) | 0.46 (0.54) | 0.67 (0.36) |
| **Meat, servings/day** | 1.32 (0.75) | 1.39 (0.70) | 1.47 (0.80) | 1.29 (0.71) | 1.38 (0.85) | 1.35 (0.79) | 1.50 (1.39) | 1.42 (0.77) |
| **Dairy products, servings/day** | 4.98 (2.59) | 4.17 (2.65) | 3.42 (2.67) | 2.01 (1.79) | 4.28 (2.49) | 3.60 (2.76) | 3.04 (2.35) | 2.16 (2.04) |
| **Alcohol, g/day** | 6.60 (9.18) | 8.26 (8.25) | 9.61 (9.15) | 11.67 (9.10) | 7.49 (8.95) | 9.66 (9.83) | 10.93 (9.89) | 12.71 (9.36) |
| **Monoansaturated to saturated fat ratio** | 0.71 (0.09) | 0.77 (0.12) | 0.82 (0.13) | 0.87 (0.15) | 0.72 (0.11) | 0.76 (0.12) | 0.82 (0.13) | 0.87 (0.12) |

**Table S3. Baseline characteristics of RA cases with (n=1119) or without (n=592) rheumatoid factor (RF) participating to the EIRA study, by categories of the Mediterranean-diet score.**

|  | **RF positive** | | | | | | | **RF negative** | | | |
| --- | --- | --- | --- | --- | --- | --- | --- | --- | --- | --- | --- |
| **Med Score** | **0-2** | | **3** | | **4-5** | | **6-9** | **0-2** | **3** | **4-5** | **6-9** |
| **N** | 238 | 232 | | 401 | | 248 | | 113 | 90 | 226 | 163 |
| **Vegetables,servings/day** | 1.65 (0.97) | 2.44 (1.61) | | 3.29 (1.84) | | 4.45 (2.06) | | 1.51 (1.03) | 2.33 (1.49) | 3.19 (1.73) | 4.67 (2.23) |
| **Legumes, servings/day** | 0.10 (0.15) | 0.14 (0.14) | | 0.23 (0.24) | | 0.39 (0.30) | | 0.08 (0.10) | 0.14 (0.19) | 0.22 (0.25) | 0.37 (0.37) |
| **Fruit and nuts, servings/day** | 0.76 (0.70) | 1.11 (1.00) | | 1.62 (1.19) | | 2.46 (1.49) | | 0.75 (0.72) | 1.13 (1.53) | 1.59 (1.29) | 2.67 (1.79) |
| **Cereals, servings/day** | 2.06 (1.75) | 2.81 (1.93) | | 2.81 (1.90) | | 3.35 (1.75) | | 2.00 (1.77) | 2.26 (1.66) | 3.07 (1.92) | 3.37 (1.87) |
| **Fish, servings/day** | 0.27 (0.17) | 0.37 (0.22) | | 0.50 (0.72) | | 0.67 (0.32) | | 0.28 (0.15) | 0.35 (0.24) | 0.52 (0.33) | 0.71 (0.35) |
| **Meat, servings/day** | 1.27 (0.75) | 1.29 (0.70) | | 1.31 (0.80) | | 1.30 (0.78) | | 1.19 (0.65) | 1.25 (0.60) | 1.32 (0.66) | 1.16 (0.70) |
| **Dairy products, servings/day** | 4.57 (2.48) | 3.70 (2.47) | | 3.07 (2.50) | | 2.05 (1.90) | | 4.74 (2.05) | 3.23 (2.52) | 3.29 (2.44) | 2.42 (2.21) |
| **Alcohol, g/day** | 4.60 (7.06) | 6.45 (7.41) | | 7.27 (8.08) | | 7.65 (6.60) | | 6.25 (9.43) | 8.15 (10.23) | 7.32 (7.80) | 9.25 (8.22) |
| **Monoansaturated to saturated fat ratio** | 0.70 (0.09) | 0.74 (0.12) | | 0.79 (0.13) | | 0.87 (0.15) | | 0.69 (0.09) | 0.76 (0.13) | 0.80 (0.14) | 0.85 (0.16) |

**Table S4. Baseline characteristics of RA cases with (n=1173) or without (n=538) anti-citrullinated protein (ACPA) participating to the EIRA study, by categories of the Mediterranean-diet score.**

|  | **ACPA positive** | | | | | | | **ACPA negative** | | | |
| --- | --- | --- | --- | --- | --- | --- | --- | --- | --- | --- | --- |
| **Med Score** | **0-2** | | **3** | | **4-5** | | **6-9** | **0-2** | **3** | **4-5** | **6-9** |
| **N** | 246 | 240 | | 423 | | 264 | | 103 | 84 | 203 | 148 |
| **Vegetables,servings/day** | 1.57 (0.96) | 2.41 (1.57) | | 3.26 (1.83) | | 4.46 (2.06) | | 1.68 (1.05) | 2.41 (1.59) | 3.25 (1.74) | 4.65 (2.23) |
| **Legumes, servings/day** | 0.10 (0.14) | 0.14 (0.16) | | 0.24 (0.25) | | 0.38 (0.28) | | 0.09 (0.13) | 0.13 (0.12) | 0.21 (0.22) | 0.37 (0.40) |
| **Fruit and nuts, servings/day** | 0.72 (0.58) | 1.03 (0.91) | | 1.63 (1.29) | | 2.50 (1.63) | | 0.86 (0.93) | 1.36 (1.68) | 1.58 (1.10) | 2.59 (1.60) |
| **Cereals, servings/day** | 2.04 (1.79) | 2.60 (1.89) | | 2.81 (1.88) | | 3.37 (1.78) | | 2.07 (1.68) | 2.71 (1.84) | 3.11 (1.96) | 3.34 (1.82) |
| **Fish, servings/day** | 0.27 (0.17) | 0.37 (0.22) | | 0.51 (0.71) | | 0.67 (0.32) | | 0.29 (0.15) | 0.35 (0.25) | 0.50 (0.29) | 0.70 (0.36) |
| **Meat, servings/day** | 1.28 (0.78) | 1.30 (0.69) | | 1.26 (0.77) | | 1.27 (0.74) | | 1.17 (0.58) | 1.21 (0.60) | 1.41 (0.70) | 1.21 (0.77) |
| **Dairy products, servings/day** | 4.67 (2.51) | 3.53 (2.47) | | 3.08 (2.47) | | 2.15 (1.91) | | 4.55 (1.91) | 3.73 (2.54) | 3.28 (2.50) | 2.23 (2.23) |
| **Alcohol, g/day** | 4.82 (7.11) | 6.86 (8.44) | | 6.93 (7.60) | | 7.70 (6.19) | | 5.88 (9.66) | 7.19 (7.91) | 8.03 (8.69) | 9.67 (9.00) |
| **Monoansaturated to saturated fat ratio** | 0.70 (0.09) | 0.74 (0.12) | | 0.79 (0.13) | | 0.87 (0.15) | | 0.69 (0.09) | 0.74 (0.12) | 0.80 (0.14) | 0.86 (0.15) |

**Table S5. Odds ratios of rheumatoid arthritis by categories of the Mediterranean-diet score among participant of the EIRA study, stratified by smoking status (RA cases n=1594, Controls n=3367)**

|  |  | **Mediterranean-diet score** | | | |
| --- | --- | --- | --- | --- | --- |
|  |  | **0-2** | **3** | **4-5** | **6-9** |
| **Never smokers** | |  |  |  |  |
|  | N | 106/253 | 110/296 | 229/716 | 160/522 |
|  | OR adjusted | Ref | 0.91 (0.66-1.26) | 0.80 (0.61-1.06) | 0.76 (0.56-1.03) |
| **Current smokers** | |  |  |  |  |
|  | N | 125/122 | 92/116 | 132/210 | 60/95 |
|  | OR adjusted | Ref | 0.76 (0.52-1.11) | 0.63 (0.45-0.60) | 0.62 (0.40-0.95) |
| **Former smokers** | |  |  |  |  |
|  | N | 98/142 | 99/181 | 228/401 | 155/313 |
|  | OR adjusted | Ref | 0.85 (0.59-1.23) | 0.88 (0.64-1.21) | 0.79 (0.56-1.10) |

**Table S6. Odds ratios of rheumatoid arthritis by categories of the Mediterranean-diet score among participant of the EIRA study, stratified by HLA-DRB1 shared epitope allele (SE) RA cases n=1426, Controls n=1559)**

|  |  | **Mediterranean-diet score** | | | |
| --- | --- | --- | --- | --- | --- |
|  |  | **0-2** | **3** | **4-5** | **6-9** |
| **With SE** | |  |  |  |  |
|  | N | 226/104 | 204/141 | 357/319 | 257/270 |
|  | OR adjusted | Ref | 0.71 (0.51-0.99) | 0.59 (0.44-0.79) | 0.60 (0.44-0.82) |
| **Without SE** | |  |  |  |  |
|  | N | 65/98 | 70/129 | 149/279 | 98/219 |
|  | OR adjusted | Ref | 0.92 (0.59-1.44) | 1.01 (0.68-1.50) | 0.90 (0.59-1.39) |

**Figure S1. Overview of patient exclusions. EIRA, Epidemiological Investigation of Rheumatoid Arthritis; FFQ, Food Frequency Questionnaire; RA, rheumatoid arthritis.**

N=21 incomplete FFQ

**N=1742**

**N=3667 matched controls**

**N=1721 RA cases included in this study**

**EIRA inclusion from November 2005 to September 2014**
